# Revealing extraordinary tensile plasticity in layered Ti-Al metal composite

**DOI:** 10.1038/srep38461

**Published:** 2016-12-05

**Authors:** M. Huang, G. H. Fan, L. Geng, G. J. Cao, Y. Du, H. Wu, T. T. Zhang, H. J. Kang, T. M. Wang, G. H. Du, H. L. Xie

**Affiliations:** 1 School of Materials Science and Engineering, Harbin Institute of Technology, Harbin, 150001, P. R. China; 2 School of Materials Science and Engineering, Harbin University of Science and Technology, Harbin, 150001, P. R. China; 3 School of Materials Science and Engineering, Dalian University of Technology, Dalian, 116000, P. R. China; 4 Shanghai Synchrotron Radiation Facility, Shanghai Institute of High Energy Applied Physics, Shanghai, 200000, P. R. China

## Abstract

Layered Ti-Al metal composite (LMC) fabricated by hot-pressing and hot-rolling process displays higher ductility than that of both components. In this paper, a combination of digital image correlation (DIC) and *X*-ray tomography revealed that strain delocalization and constrained crack distribution are the origin of extraordinary tensile ductility. Strain delocalization was derived from the transfer of strain partitioning between Ti and Al layer, which relieved effectively the strain localization of LMC. Furthermore, the extensive cracks of LMC were restricted in the interface due to constraint effect. Layered architecture constrained the distribution of cracks and significantly relieved the strain localization. Meanwhile, the transfer of strain partitioning and constrained crack distribution were believed to inhibit the strain localization of Ti and change the deformation mechanisms of Ti. Our finding enriches current understanding about simultaneously improving the strength and ductility by structural design.

High strength of metals is always required to reduce the weight of transportation vehicles to improve their energy efficiency. Good ductility is also desired because it can prevent catastrophic failure during service. However, strengthening is usually accompanied by a drop in ductility^1^. The strength-ductility trade-off is hard to be overcome in the class of metals/alloys, such as casting iron^2^ and aluminum alloys^3^, etc. In general, the strength of metals/alloys increases with decreasing grain size according to the Hall-Petch relationship^4^. However, when the grain size reduces to the nanoscale, the ductility will decrease to a low level^5^. For example, bulk nano-grained metals fabricated by severe plastic deformation (SPD) methods exhibit high strength but low ductility^4^,^5^,^6^. For achieving strength-ductility combination, several of approaches have been reported in recent decades, for example control of second phase and grain size distribution^7^,^8^, etc. In addition, composites provide a new insight to optimize overall properties by structural design of component phases, such as network^9^, bi-continuous^10^, layered^11^,^12^ and gradient^13^ structure. In particular, layered composites potentially achieve a combination of high strength and ductility by layered structural design^14^,^15^,^16^,^17^.

It has been reported that the deformation of layered composites is related to the interface constraint, which transfers the load and redistributes the stress during the deformation^18^,^19^. The constraint effect, which mutually affects the deformation of either component, has been widely investigated^20^,^21^. For instance, the brittle martensite in a brittle/ductile multilayered steel can withstands a non-localized strain of more than 20% beyond its limit^18^, indicating that brittle phase can bear larger plastic deformation through the structure design. The abnormal behavior has been well explained by the mechanism of strain partitioning during the deformation of multilayered steel^22^,^23^. And the failure of brittle martensite in the brittle/ductile multilayered steels has been delayed by the surrounding ductile phase^24^,^25^,^26^. Therefore, the direct visualization of strain evolution is useful to understand the deformation mechanism of layered composites. In recent years, DIC as the advanced characterization technique of strain has been applied to reveal the abnormal phenomenon of strain capacity^18^,^27^. The aim of this paper is to provide quantitative analysis of local strain distribution by DIC, and further to reveal the good ductility of layered composites.

In this work, layered Ti-Al metal composite (LMC) was prepared by hot-press and multi-pass hot-rolling process of commercially pure Ti and Al sheets. The deformation behavior of LMC was analyzed during *in-situ* tensile test to reveal the effect of local strain distribution on mechanical properties using DIC. In addition, the distribution of cracks in fractured LMC was studied by *X*-ray tomography to reveal the effect of constraint effect on crack initiation and propagation.

## Results

### Microstructure characterization

**Figure 1:**
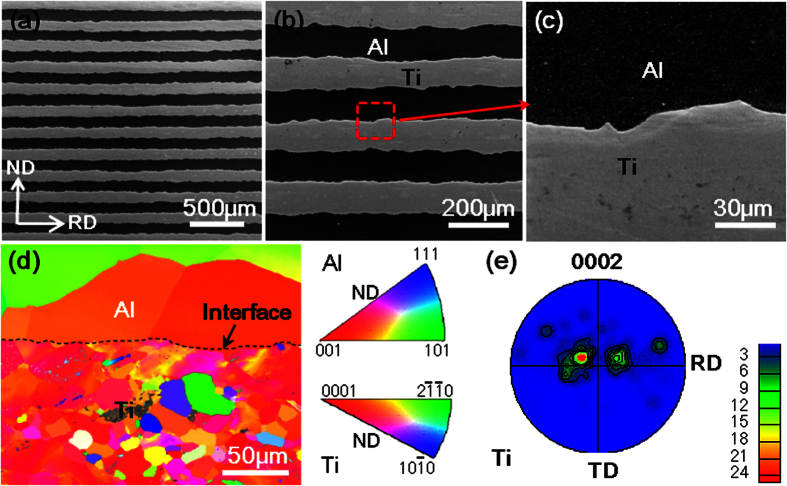
The LMC fabricated by hot pressing and hot rolling with an individual layer thickness of 100 μm for both Ti and Al; (**a,b** and **c**) the microstructure; (**d**) inverse pole figure for normal direction (ND); (**e**) (0001) pole figure for Ti in LMC.

The microstructure of LMC before tensile test was shown in Fig. 1a,b and c. LMC with an individual layer thickness of 100 μm was successfully fabricated by hot pressing and hot rolling, and the interface between Ti and Al presented a roughness of 10 μm due to multi-pass hot-rolling process. High magnification image displays well interfacial bonding. After rolling and annealing process, the Ti layer exhibited a strong basal texture of <0001>//ND, whereas the Al layer demonstrated the typical recrystallized feature and had no obvious preferred orientation, as shown in Fig. 1d and e.

### Mechanical properties

**Figure 2:**
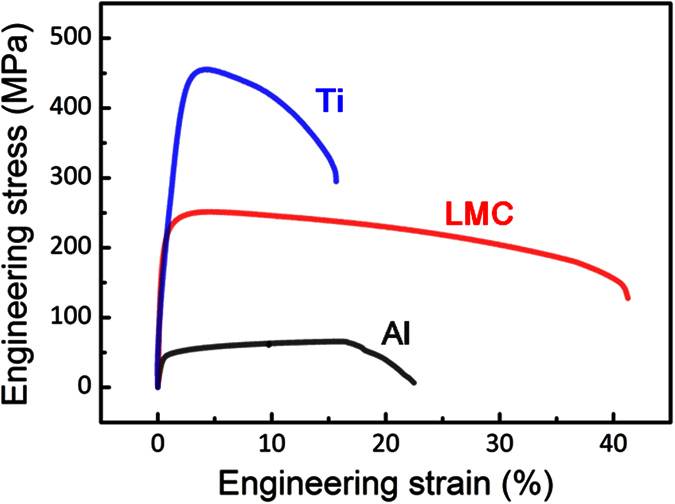
Engineering stress-strain curves of LMC, Ti and Al with the same processing route. The loading direction is parallel to transverse direction (TD).

Figure 2 compared the tensile properties of component Ti, component Al and LMC. The yield strength (0.2% offset) of LMC reached a magnitude of 250 MPa, which agrees well with the rule of mixture (ROM)^28^ (the calculated process was provided in the supplementary materials). Interestingly, the ductility of LMC (elongation of 41%) presented an abnormal behavior: higher than either component (Ti or Al). This concluded that the improvement of tensile plasticity in LMC was attributed to the unique layered structural design, and then DIC technique was introduced to study the effect of the layered structure on the local strain evolution of LMC.

### The strain distribution measured by DIC

**Figure 3:**
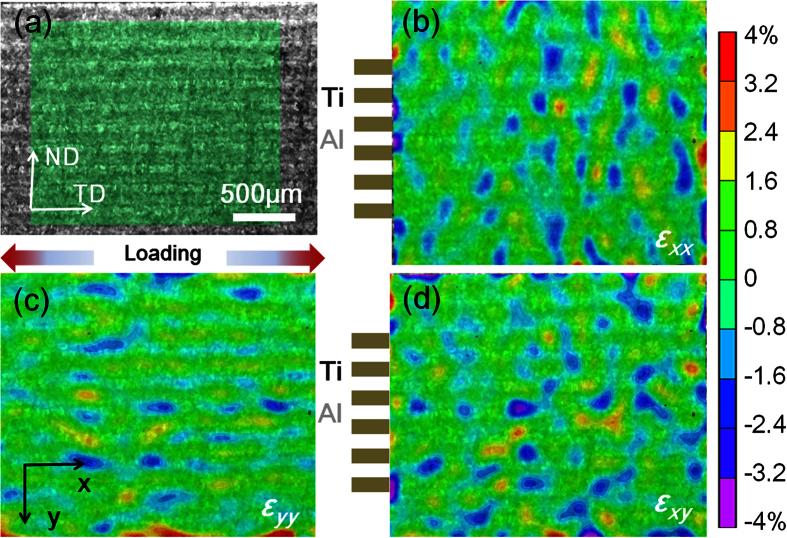
Strain distribution images of LMC obtained by DIC at a macro strain level of 4.0%. (**a**) The measured region marked by DIC; local strain maps in the (**b**) ***ε***_***xx***_, (**c**) ***ε***_***yy***_ and (**d**) ***ε***_***xy***_.

The strain distributions measured by DIC at a macro strain level of 4.0% were displayed in Fig. 3. The loading direction was parallel to the *x* direction (see Fig. 3a). Local strain maps in the *x* direction (*ε*_*xx*_), the *y* direction (*ε*_*yy*_) and the shear direction (*ε*_*xy*_) were shown in Fig. 3b,c and d, respectively. The *ε*_*xx*_ along tensile direction displays evident strain partition with compression and tension strain regions (Fig. 3b). This suggests that at a low strain level strain localization emerged. Figure 3c illustrates that the strain state of *ε*_*yy*_ in LMC had certain regularity and periodicity. Along the *y* direction, the compression strain in the softer layer (Al) was always accompanied by the tension strain in the hard layers (Ti). The shear strain values were relatively low at current macro strain level. Nevertheless, *ε*_*xy*_ was mainly distributed in the interface between Ti and Al (Fig. 3d).

### The evolution of the local strains during deformation

**Figure 4:**
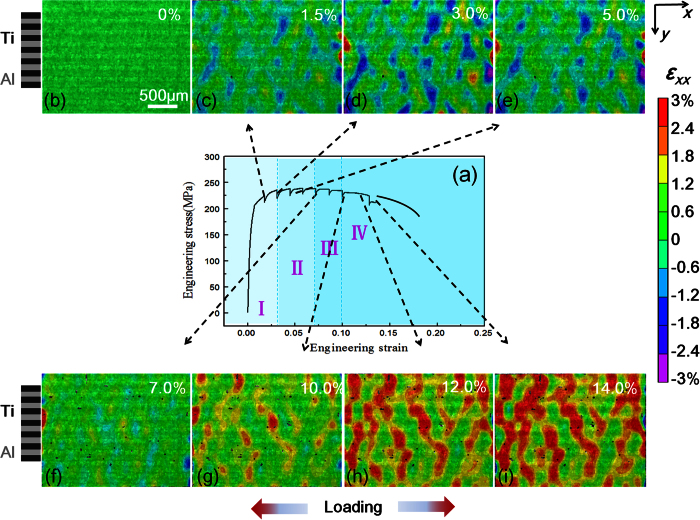
The evolution of *ε*_*xx*_ strains along the loading direction at different macro strain levels. (**a**) corresponding stress-strain curves of LMC; macro strains at (**b**) 0; (**c**) 1.5%; (**d**) 3.0%; (**e**) 5.0%; (**f**) 7.0%; (**g**) 10.0%; (**h**) 12.0%; (**i**) 14.0%.

The engineering stress-strain curve of LMC was shown in Fig. 4a. The serration of the stress-strain curve appeared due to the relaxation of stress during capturing the micrographs. However, when the macro strain was too large, the surface of sample would be destroyed due to severe deformation, and DIC analysis became difficult. The *in-situ* test was stopped at a macro strain level of 14%.

To reveal the local strain evolution during tensile deformation, the *ε*_*xx*_ distribution were tracked at different macro strain levels (1.5%, 3.0%, 5.0%, 7.0%, 10.0%, 12.0% and 14.0%), as shown in Fig. 4b~i. According to the difference of local strain distribution, the deformation of LMC was divided into four stages. **Stage I (0~3.0%)**: with the increase of macro strain the heterogeneity of strain distribution gradually appeared, and there were significant strain localization with distinct compression and tension strain regions. It is apparent that strain localization was originally detected at the interface (between Ti and Al) and the softer Al layers, rather than Ti layer (Fig. 4b,c and d); **Stage II (3.0~7.0%)**: this stage was a process of strain redistribution. With macro strain increasing, the compression strain along the *x* direction disappeared, the regions of strain localization were alleviated and redistributed, and then strain distribution was more uniform. As a result, strain localizations were released (Fig. 4e and f); **Stage III (7.0~10.0%)**: the harder Ti layers started to undertake more plastic strains and presented the trend of strain localization (Fig. 4g); **Stage IV (10.0%~)**: apparent strain partitions transferred across the interfaces from Ti layers to Al layers (Fig. 4h and i). Local strains were not concentrated in a single layer or at the interface, but penetrated into the adjacent layers and spread over wider regions. The four stages indicate that the evolution of local strains in LMC was a process of strain delocalization and strain redistribution.

**Figure 5:**
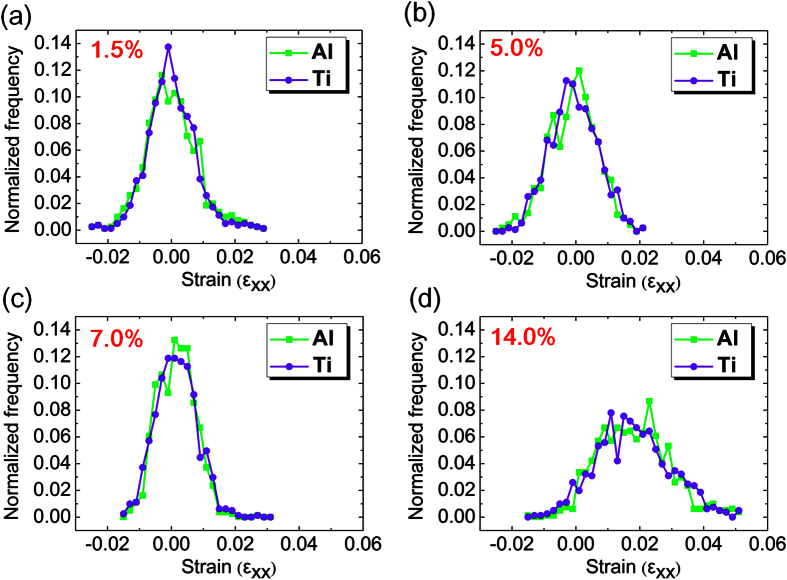
Strain partitioning of component (Ti and Al) at different macro strains of (**a**) 1.5%; (**b**) 5.0%; (**c**) 7.0%; (**d**) 14.0%.

Figure 5 illustrates quantitative statistics about strain partitioning of components (Ti and Al) along *x* direction at different macro strain levels, respectively, corresponding to the above four stages. At the early deformation stage, soft Al layers preferentially yielded and hard Ti layers imparted a reverse compression stress to Al layers due to constraint effect. Therefore, at stage I, compared with Ti layers, the Al layers partially withstood a compression strain due to early incompatible deformation (Fig. 5a). At stage II, there was significant strain localization, and the majority of *ε*_*xx*_ local strains were mainly accommodated by Al layer (Fig. 5b). It is also concluded that local strain concentration preferentially occurred in softer Al layer. At a macro strain level of 7.0%, the curves of local strain distribution become narrow, and the deformation of Ti and Al layers is more homogeneous (Fig. 5c). This indicated that strain localization of Al layers were released by the redistribution of strain at stage II. Nevertheless, at stage III and IV with a higher macro strain level up to 14.0%, both Ti and Al simultaneously underwent the tensile strain due to the transfer of strain localization (Fig. 5d). Note that the curves moved to the right as a whole, indicative of plastic deformation in both layers. The deformation in a wider dimension was believed to enhance their average strain level by forming a strain gradient distribution in the adjacent regions. Therefore, layered architecture redistributed the local strain, and suppressed the strain localization of single component layer.

### Fracture behavior based on X-ray tomography

**Figure 6:**
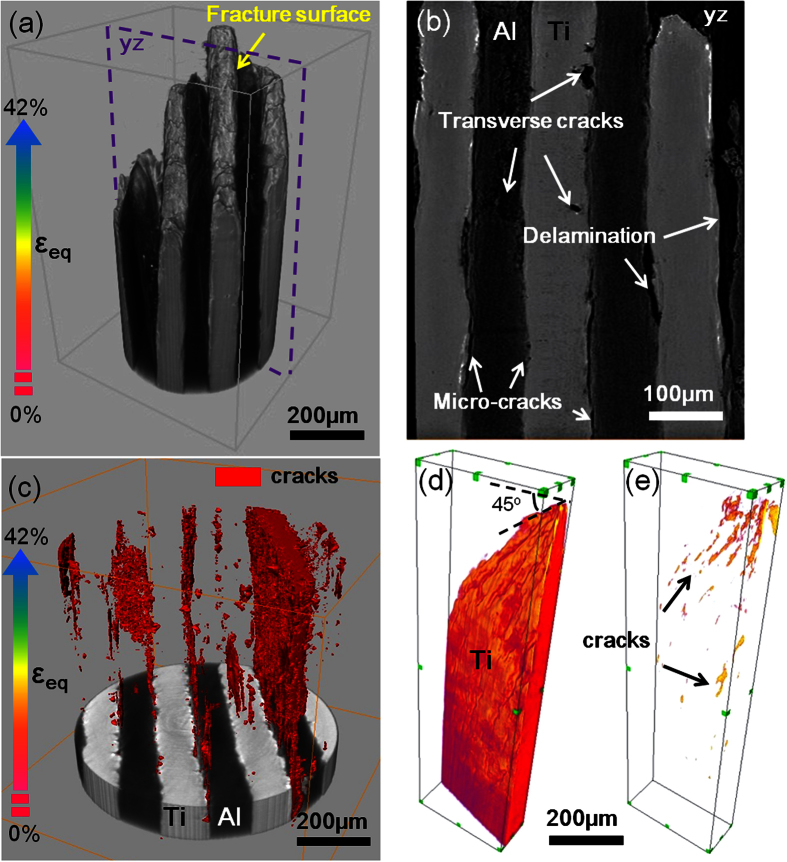
The 3D rendering of fractured LMC. (**a**) Fractured composite; (**b**) 2D tomographic slice of yz plane in (**a**), (**c**) 3D distribution of cracks in LMC; the 3D rendering of abstracted Ti: (**d**) fractured abstracted Ti; (**e**) 3D distribution of cracks in abstracted Ti.

In order to identify and showcase damage behavior of LMC and component Ti abstracted from LMC, the 3D renderings of cracks in the fractured sample were illustrated in Fig. 6. To establish a relationship between different positions of fractured sample and macro tensile strain, a new definition of equivalent strain (*ε*_*eq*_) was introduced. The *ε*_*eq*_ at different positions in the fractured sample can be estimated from the gauge sectional area by *ε*_*eq*_ = *ln(S*_*0*_*/S)* (where *S*_*0*_ is the original gauge sectional area and *S* is the gauge sectional area)^29^.

The 3D morphology of LMC after tensile fracture was shown in Fig. 6a. Fractured composite displayed a severely plastic deformation with a shear fracture. Close inspection of damage in LMC shows that there were three major types of damage (Fig. 6b): (i) locally interfacial micro-cracks between Ti and Al layers; (ii) transverse cracks, which were restrained by Ti layer without sequential propagation during deformation and finally appeared in Al layer approaching the region of fracture; (iii) delamination, which was formed after whole interface was stripped at a high strain level. Figure 6c shows that the damage of LMC developed progressively from the low *ε*_*eq*_ to high *ε*_*eq*_. At a low *ε*_*eq*_ level, a few micro-cracks occurred within the interface due to early incompatible deformation. As the *ε*_*eq*_ increases, the cracks propagated along the interface by inheriting sequent damage elsewhere, but we found that the cracks were steadily restricted in the interface without transverse propagation. Once the interface delamination occurred, constraint effect would disappear locally. Without constraint effect, the Al layers quickly necked and failed at a higher *ε*_*eq*_ level, resulting in the ultimate fracture of LMC.

Nevertheless, for abstracted Ti, the micro-cracks in three dimensions are intensively distributed in a necking region with a 45° shear fracture (supplementary Fig. S1), indicating rapid initiation and propagation of cracks in abstracted Ti (Fig. 6d and e).

**Figure 7:**
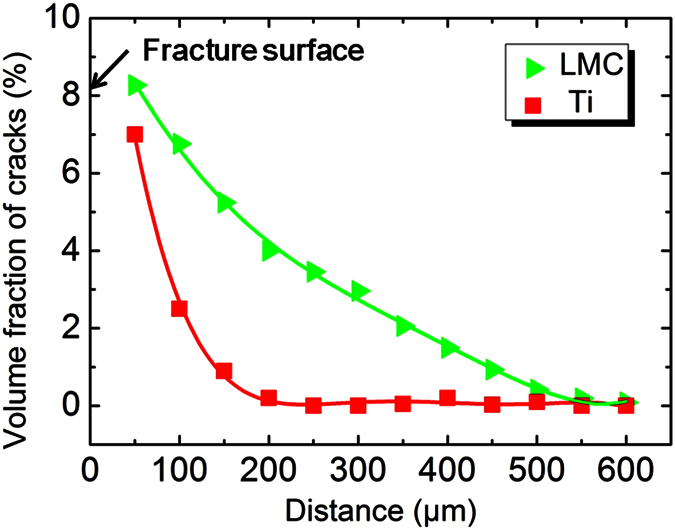
Quantitative analysis of the distribution of cracks volume fraction as a function of distance away from the fracture surface.

Figure 7 illustrates the quantitative analysis of crack volume fraction with increasing distance from the fracture surface. The volume fraction of cracks was the largest at the fracture surface and decreased monotonically with increasing the distance from the fracture surface. It is found that the crack volume fraction in LMC decreased slower than that in the abstracted Ti. This means that constraint effect of layered architecture relieved the propagation rate of crack and changed the distribution of cracks in LMC, which made LMC consume more fracture energy and display superior fracture toughness.

## Discussion

### The transfer of strain partitioning

The ductility of LMC is higher than either component Ti or Al. The extraordinary tensile plasticity of LMC is ascribed to the transfer of strain localization. The local strain evolution of LMC presents a process of strain delocalization which is related to crystal texture, component property and layered structural design. At the initial deformation stage, the deformation incompatibility between Ti layer and Al layer occurs and the large amount of strain is accommodated by the interface and soft Al layer^30^,^31^,^32^. The mismatched stress caused by the incompatible deformation can gradually be remedied by back stress^21^, which is experimentally supported by more uniform deformation at stage II. In fact, the harder Ti layers are easier to present strain localization at a high strain level due to the strong basal texture of <0001>//ND. Nevertheless, at a high macro strain level, strain localization of Ti layers gradually transfer to Al layers. Consequently, strain compatibility between Ti layer and Al layer is significantly enhanced by layered architecture. Additionally, the redistribution of strain effectively relieves the strain localization of Ti layer, and it promotes adjacent Ti regions to assume more plastic deformation. Therefore, constraint effect of layered architecture is believed to change the deformation mechanisms of Ti by transferring localized strain.

### Effect of constraint effect on the deformation mechanisms of Ti

**Figure 8:**
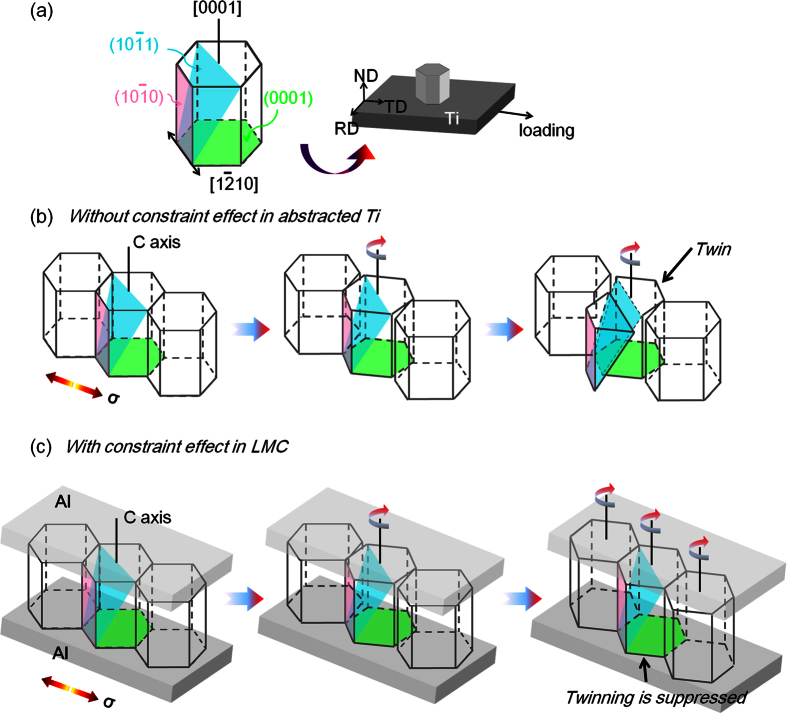
Schematic drawing of plastic deformation. (**a**) Ti crystal; (**b**) the abstracted Ti display the easily activated twins along c axis; (**c**) the Ti in LMC shows twinning is suppressed.

The slip and twinning are the main plastic deformation mechanisms of hcp Ti (α-Ti) at room temperature^33^,^34^. It has been reported that in commercial pure Ti the imposed shear strain was accommodated by compression twins^33^. Component Ti in LMC shows strong basal texture <0001>//ND presented in section 3.1, as schematically shown in Fig. 8a. The prismatic slip, by contrast, is easier to be activated than basal slip, and most of the <11–20> are located in the RD-TD plane of Ti layer. Therefore, the prismatic slip withstands plastic deformation by the rotation of Ti grains around the *c* axis, and it could not cause any thinning of Ti layer along the ND direction. Under the current crystallographic textures, the necking of Ti layer must be caused by the *c* axis deformation provided by compression twins.

For abstracted Ti, once the prismatic slip with the rotation of Ti grains hardly withstands more plastic deformation in loading direction, the *c* axis deformation would be increasingly accommodated by compression twinning (Fig. 8b). As a result, the necking and fracture of Ti would rapidly occur, and also explain the reason why almost cracks appear near the fracture of abstracted Ti. However, for Ti in LMC, the compression twinning is restrained due to the constraint effect of layered architecture, and then more tensile strains are continuously accommodated by adjacent grains through the prismatic slip - grain rotation (Fig. 8c). Thus, constraint effect of layered architecture relieves strain localization of Ti by transferring strain to the adjacent regions.

### Effect of constraint effect on crack distribution

Analysis of fracture behavior in LMC in section 3.5 shows that the disappearance of constraint effect is derived from the delamination, which is considered as the cause of the reduced plasticity. The delamination can be prevented at a low strain level due to strong interfacial bonding strength by hot rolling. At the early deformation stage, interfacial mismatched stress caused by incompatible deformation gradually increases during the deformation. If the mismatched stress exceeds the bonding strength, the interface will crack and form the early micro-cracks. It is interesting to note that the micro-cracks are restricted in the interface without transverse propagation during the tensile deformation. On one hand, these crack-tips are blunted by Ti and Al layers, which relieve stress concentration of crack-tip and suppress the transverse propagations of cracks. On the other hand, due to the introduction of layered architecture, theoretically uniaxial tensile stress is converted to multiaxial stress states^35^. Compared with Ti layers, Al layers present a compression strain at the *ε*_*yy*_strain map in Fig. 5c, and the Al layer provide an outward force along the *y* direction to Ti layer, which delays the necking of Ti layer^36^. If crack-tip stress is enough to destroy the interface bonding, these cracks propagate along the interface to suppress localized damage by inheriting sequent damage elsewhere. Once these interfacial cracks propagate to form the delamination, constraint effect disappears locally. Then local large transverse cracks are easy to occur, resulting in the damage of LMC.

In order to determine crack morphologies at various strain stages and further to establish the relationship between deformation behavior of LMC and crack evolution, it assumes that the *ε*_*eq*_is equal to tensile macro strain of LMC^29^. The reasonability of the assumption is experimentally supported by an *in-situ* tensile test based on SEM (Supplementary Part C). We compare crack morphologies at different deformation stages. It can be concluded that: **i)** at a low macro strain of 3.0%, dispersive micro-cracks appear within the interface due to early incompatible deformation, corresponding to the stage I in Fig. 4. In this stage, strain localization (Fig. 4d) and local necking (Fig. S2b) are firstly detected at the interface, and may be related to the initiation of micro-cracks; **ii)** these micro-cracks are steadily restricted in the interface and hardly propagate below a macro strain of 10.0%. The local failure of hard Ti is delayed by neighboring Al layers, presenting a redistribution process of strain partitioning, and corresponding to the stage II and III in Fig. 4; **iii)** as macro strain exceeds 10%, more local neckings of Ti layer appear, and micro-cracks propagated along the interface to form the delamination. Constraint effect of layered architecture disappeared locally, and local strains gradually transfer from Ti layer to Al layer (the stage IV in Fig. 4). Therefore, we conclude that the emergence of micro cracks does not affect the deformation behavior of LMC, and these micro-cracks are always constrained in the interface due to the constraint effect of layered architecture.

## Conclusions

The LMC fabricated by the same hot-pressing and hot-rolling process demonstrated a good ductility, being superior to any component Ti and Al. In this paper, the local stain evolution of LMC was investigated to *in situ* track the strain distribution to reveal the high ductility. Furthermore, the fracture behavior of LMC was studied using *X*-ray tomography to reveal the mechanisms of crack initiation and propagation. The conclusions were drawn as follows:

- The extraordinary tensile plasticity of LMC was ascribed to the transfer of strain localization. It is found that the advantage of the layered structural design reflected in the strain delocalization by the transference of strain partitioning from Ti to Al layer, which effectively relieved the strain localization of LMC.
- The 3D renderings of the fracture of LMC and abstracted Ti were illustrated. The extensive micro-cracks of LMC were restricted in the interface due to constraint effect of layered architecture. Layered architecture constrained the distribution of micro-cracks, and relieved the strain localization. Additionally, from the relationship between deformation behavior of LMC and crack evolution, the emergence of micro cracks does not affect the deformation behavior of LMC.
- Both the transference of strain localization and restricted crack distribution were believed to inhibit the strain localization of Ti in LMC and change the deformation mechanisms of Ti in LMC. For abstracted Ti, the c axis deformation was easy to be activated by twinning. However, the c axis deformation of Ti in LMC was restrained due to the constraint effect of layered architecture. More tensile strains were continuously accommodated by adjacent grains. Layered architecture relieved the strain localization by transferring strain to the adjacent regions.

## Materials and Methods

### Materials

Commercially pure titanium sheets TA1 with an original thickness of 200 μm and pure aluminum sheets 1060 with an original thickness of 200 μm were selected as raw materials. The sheets were cut into 100 × 100 mm^2^ squares. The schematics of the fabrication processing of LMC were shown in the supplementary materials. Firstly, Ti sheets and Al sheets were chemically etched in 10 vol.% HF solution and 10 wt.% NaOH solution, respectively, in order to remove surface oxide layer. Then, these sheets were alternately placed and processed in vacuum at 500 °C for 1 h with a pressure of 40 MPa to obtain well interface bonding. Finally, LMC was rolled to a total thickness reduction of 50% after 6 rolling passes and annealed at 500 °C for 10 minutes after each rolling pass. The details of rolling conditions have been mentioned elsewhere^37^.

### Microstructure characterization

The microstructure of LMC was detected using backscattered electron (BSE) mode in a scanning electron microscope (SEM, HITACHI S-570), and scanning region was the rolling direction - normal direction (RD-ND) plane. The grain orientation was observed by a scanning electron microscope (SEM, FEI HELIOS Nanolab 600i) equipped with an HKL electron backscatter diffraction (EBSD) facility. The EBSD specimens were polished by ion beam lithography on the RD-ND section. The step size of 0.2 μm was selected to acquire EBSD mapping with the region of 160 × 140 μm^2^.

### Tensile test

The tensile tests were carried out at room temperature (RT) using an Instron-1186 Universal Testing Machine with a strain rate of 5 × 10^–4^ s^−1^. To compare with the mechanical properties of LMC, individual Ti was abstracted from LMC using NaOH solution soaking, and individual as-rolled Al was prepared by the same rolling process from 200 μm to 100 μm. Dog-bone shaped tensile specimens were prepared by electrical discharge machining with a thickness of 2 mm, a width of 5 mm and the gauge length of 18 mm. Three tensile specimens were tested for each type of materials.

### *In-situ* tensile test based on optical microscope

An interrupted tensile experiment was carried out using a Kammrath-Weiss micro-tensile stage, placed in the optical microscope (OM). For tensile testing half dog-bone shaped specimens were prepared to match with the dimension of the micro-tensile stage. The experimental set-up and geometry dimensions of the tensile sample for *in-situ* tensile test were displayed in the supplementary materials. In this work, the practical speed of displacement was about 5 μm/s which corresponded to a conventional strain rate of 5 × 10^−4^ s^−1^.

Serial high-resolution snapshots were obtained at successive deformation stages. The image processing was performed in a reduced zone of specimen surface which covered a surface of 500 × 420 pixels, corresponding to the red area (see Supplementary Fig. S4b). Small scratches of specimen surface were artificially made by mechanically polishing the surface using the 4000-grit sandpaper. The difference in the positions of small scratches on surface of sample before and after deformation was used to quantify the local strain^38^. Additionally, the strain distributions were analyzed using the VIC-2D software.

### Fracture behavior based on X-ray tomography

The fracture behavior of LMC was studied using X-ray tomography in the beamline station BL13W1 of Shanghai Synchrotron Radiation Facility (SSRF). The size of specimen and the test parameters are shown in the supplementary materials. The Ti specimen was abstracted from LMC using NaOH solution soaking. To showcase the distribution of cracks, the volume fraction of cracks with increasing distance from the fracture surface was estimated by *ƒ*_*crack*_ = *V*_*crack*_*/V*_*total*_ (where *V*_*crack*_ is the volume of cracks and *V*_*total*_ is the volume of LMC or abstracted Ti). The detailed method to determine the crack volume is introduced in supplementary materials. The quantitative statistics of crack volume fraction was calculated once every 50 μm. The fracture mechanisms of LMC and component Ti were analyzed.

## Additional Information

**How to cite this article**: Huang, M. *et al*. Revealing extraordinary tensile plasticity in layered Ti-Al metal composite. *Sci. Rep.* **6**, 38461; doi: 10.1038/srep38461 (2016).

**Publisher's note:** Springer Nature remains neutral with regard to jurisdictional claims in published maps and institutional affiliations.

## Supplementary Material

Supporting Information

## References

[b1] Lu K. The future of metal. Science 328, 319–320 (2010). 20395503 10.1126/science.1185866

[b2] Mizushima T., Ikarashi K., Yoshida S., Makino A. & Inoue A. Soft magnetic properties of ring shape bulk glassy Fe–Al–Ga–P–C–B–Si alloy prepared by copper mold casting. Mater. Trans., JIM 40, 1019–1022 (1999).

[b3] Tanihata H., Sugawara T., Matsuda K. & Ikeno S. Effect of casting and homogenizing treatment conditions on the formation of Al–Fe–Si intermetallic compounds in 6063 Al–Mg–Si alloys. J. Mater. Sci. 34, 1205–1210 (1999).

[b4] Li S., Sun L. & Wang Z. A model of Hall-petch relationship in nanocrystalline materials. Nano. Mater. 2, 653–661 (1993).

[b5] Meyers M. A., Mishra A. & Benson D. J. Mechanical properties of nanocrystalline materials. Prog. Mater Sci. 51, 427–556 (2006).

[b6] Zhang P. . Optimizing strength and ductility of Cu–Zn alloys through severe plastic deformation. Scr. Mater. 67, 871–874 (2012).

[b7] Liu G. . Nanostructured high-strength molybdenum alloys with unprecedented tensile ductility. Nat. Mater. 12, 250–344 (2013). 10.1038/nmat3544 23353630

[b8] Zhu Y. T. & Liao X. Nanostructured metals: retaining ductility. Nat. Mater. 3, 351–352 (2004). 15173850 10.1038/nmat1141

[b9] Huang L. J. . Tailoring a novel network reinforcement architecture exploiting superior tensile properties of *in situ* TiBw/Ti composites. Mater. Sci. Eng., A 545, 187–193 (2012).

[b10] Peng H. X., Fan Z. & Evans J. R. G. Bi-continuous metal matrix composites. Mater. Sci. Eng., A 303, 37–45 (2001).

[b11] Deville S., Saiz E., Nalla R. K. & Tomsia A. P. Freezing as a path to build complex composites. Science 311, 515–518 (2006). 16439659 10.1126/science.1120937

[b12] Launey M. E. . A novel biomimetic approach to the design of high-performance ceramic-metal composites. J. R. Soc. Interface 7, 741–753 (2010). 19828498 10.1098/rsif.2009.0331 PMC2874234

[b13] Rodriguez-castro R., Wetherhold R. C. & Kelestemur M. H. Microstructure and mechanial behavior of functionally graded Al359_SiCp composites. Mater. Sci. Eng., A 323, 445–456 (2002).

[b14] Pang J. C. . Mechanical properties of Ti–(SiCp/Al) laminated composite with nano-sized TiAl3 interfacial layer synthesized by roll bonding. Mater. Sci. Eng., A 582, 294–298 (2013).

[b15] Tanaka K. & Mori T. The hardening of crystals by non-deforming particles and fibers. Acta. Metall. 18, 931–939 (1970).

[b16] Ortiz C. & Boyce M. C. Bioinspired structural materials. Science 319, 1053–1054 (2008). 18292331 10.1126/science.1154295

[b17] Chaudhari G. P. & Acoff V. Cold roll bonding of multi-layered bi-metal laminate composites. Compos. Sci. Technol. 69, 1667–1675 (2009).

[b18] Lhuissier P., Inoue J. & Koseki T. Strain field in a brittle/ductile multilayered steel composite. Scr. Mater. 64, 970–973 (2011).

[b19] Li Y. P. & Zhang G. P. On plasticity and fracture of nanostructured Cu/X (X=Au, Cr) multilayers: The effects of length scale and interface/boundary. Acta. Mater. 58, 3877–3887 (2010).

[b20] Na H., Nambu S., Ojima M., Inoue J. & Koseki T. Strain localization behavior in low-carbon martensitic steel during tensile deformation. Scr. Mater. 69, 793–796 (2013).

[b21] Wu X. L. . Heterogeneous lamella structure unites ultrafine-grain strength with coarse-grain ductility. Proc. Natl. Acad. Sci. USA 112, 14501–14505 (2015). 26554017 10.1073/pnas.1517193112 PMC4664339

[b22] Barabash R. I. . Interphase strain gradients in multilayered steel composite from microdiffraction. Metall. Mater. Trans. A 45, 98–108 (2013).

[b23] Ojima M. . Stress partitioning behavior of multilayered steels during tensile deformation measured by *in situ* neutron diffraction. Scr. Mater. 66, 139–142 (2012).

[b24] Pu C. & Gao Y. Crystal plasticity analysis of stress partitioning mechanisms and their microstructural dependence in advanced steels. J. Appl. Mech. 82, 031003 (2015).

[b25] Inoue J., Nambu S., Ishimoto Y. & Koseki T. Fracture elongation of brittle/ductile multilayered steel composites with a strong interface. Scr. Mater. 59, 1055–1058 (2008).

[b26] Nambu S., Michiuchi M., Inoue J. & Koseki T. Effect of interfacial bonding strength on tensile ductility of multilayered steel composites. Compos. Sci. Technol. 69, 1936–1941 (2009).

[b27] Joo S. H. . Method for measuring nanoscale local strain in a dual phase steel using digital image correlation with nanodot patterns. Scr. Mater. 68, 245–248 (2013).

[b28] Lesuer D. R. . Mechanical behaviour of laminated metal composites. Int. Mater. Rev. 41, 169–197 (1996).

[b29] Fang T. H., Li W. L., Tao N. R. & Lu K. Revealing extraordinary intrinsic tensile plasticity in gradient nano-grained copper. Science 331, 1587–1590 (2011). 21330487 10.1126/science.1200177

[b30] Han Q., Kang Y., Hodgson P. D. & Stanford N. Quantitative measurement of strain partitioning and slip systems in a dual-phase steel. Scr. Mater. 69, 13–16 (2013).

[b31] Tasan C. C., Hoefnagels J. P. M. & Geers M. G. D. Microstructural banding effects clarified through micrographic digital image correlation. Scr. Mater. 62, 835–838 (2010).

[b32] Wang Y. M., Chen M. W., Zhou F. H. & Ma E. High tensile ductility in a nanostructured metal. Nature 419, 912–915 (2002). 12410306 10.1038/nature01133

[b33] Kim I., Kim J., Shin D. H., Liao X. Z. & Zhu Y. T. Deformation twins in pure titanium processed by equal channel angular pressing. Scr. Mater. 48, 813–817 (2003).

[b34] Kim W. J., Yoo S. J. & Lee J. B. Microstructure and mechanical properties of pure Ti processed by high-ratio differential speed rolling at room temperature. Scr. Mater. 62, 451–454 (2010).

[b35] Wu X. L., Jiang P., Chen L., Yuan F. & Zhu Y. T. Extraordinary strain hardening by gradient structure. Proc. Natl. Acad. Sci. USA 111, 7197–7201 (2014). 24799688 10.1073/pnas.1324069111 PMC4034219

[b36] Launey M. E. & Ritchie R. O. On the fracture toughness of advanced materials. Adv. Mater. 21, 2103–2110 (2009).

[b37] Du Y. . Effects of interface roughness on the annealing behaviour of laminated Ti-Al composite deformed by hot rolling. *IOP Conference Series: Materials Science and Engineering* **89**, 012–021 (2015).

[b38] Grégoire D., Loh O., Juster A. & Espinosa H. D. *In-situ* AFM experiments with discontinuous DIC applied to damage identification in biomaterials. Exp. Mech. 51, 591–607 (2011).

