# Supplementary material for: Revealing extraordinary tensile plasticity in layered Ti-Al metal composite
Source: Sci Rep. 2016 Dec 5;6:38461. doi: 10.1038/srep38461 (PMC5137039; doi:10.1038/srep38461)
Supplement: Supporting Information [file srep38461-s1.doc]

**Supporting Information:**

**Revealing extraordinary tensile plasticity in layered Ti-Al metal composite**

M. Huang^a^, G.H. Fan^a^*, L. Geng^a^, G.J. Cao^b^, Y. Du^a^, H. Wu^a^, T.T. Zhang^a^, H.J. Kang^c^, T.M. Wang^c^, G.H. Du^d^, H.L. Xie^d^

^a^ School of Materials Science and Engineering, Harbin Institute of Technology, Harbin, 150001, P. R. China

^b^ School of Materials Science and Engineering, Harbin University of Science and Technology, Harbin, 150001, P. R. China

^c^ School of Materials Science and Engineering, Dalian University of Technology, Dalian, 116000, P. R. China

^d^ Shanghai Synchrotron Radiation Facility, Shanghai Institute of High Energy Applied Physics, Shanghai, 200000, P. R. China

** Corresponding author. Tel:* *+86-451-86418836; Fax:* *+86-451-86413922*

*E-mail: ghfan@hit.edu.cn (G.H. Fan)*

***Part A Estimate of the yield strength of LMC***

The tensile test results of LMC and individual material (abstracted Ti and as-rolled Al) exhibit the difference in yield point. The yield strength of abstracted Ti displays 430 MPa (*σ_Ti_*), while as-rolled Al shows the tensile result with the yield strength of 48 MPa (*σ_Al_*). In contrast, LMC unites the yield strength of both individual Ti and Al, reaching a magnitude of 250 MPa. According to the ROM equation: *σ_LMC_ = V_Ti_×σ_Ti +_V_Al_×σ_Al_*, the yield strength of LMC (*σ_LMC_*) is calculated. There, the volume fraction of Ti and Al in LMC is about *V_Ti_* =0.52 and *V_Al_* =0.48, respectively, due to 12 layers of Ti and 11 layers of Al. Calculated related data are displayed in the Table S1. The calculated result: *σ_LMC_* = 247 MPa, is similar to the real yield strength of LMC.

**Table S1**. Related parameters about the calculation of yield strength of LMC

| Materials | Number of layers | Volume fraction (*V*) | Yield strength (*σ*, MPa) |
| --- | --- | --- | --- |
| Al | 11 | 0.52 | 48 |
| Ti | 12 | 0.48 | 430 |

***Part B Measurement of shear angle of fracture in abstracted Ti***

The shear angle of fracture in abstracted Ti was measured. To provide more experimental supports, the macro picture and 3D rendering of fractured abstracted Ti was given with the measured angle of 45°, as shown in Fig. S1.


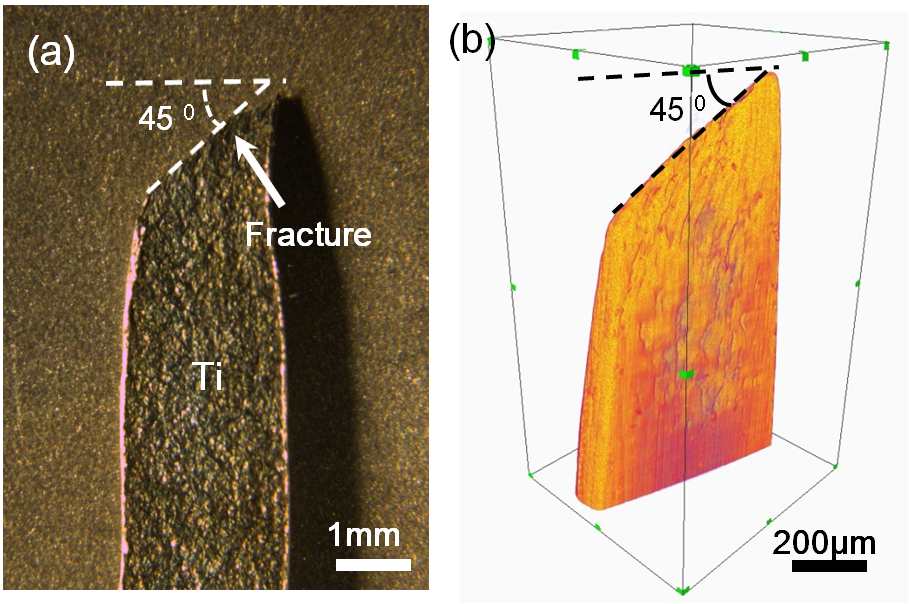


Fig. S1 (a) The macro picture of fractured abstracted Ti; (b) the 3D rendering of fractured abstracted Ti.

***Part C Crack morphologies at different strain stages***

To determine crack morphologies at various strain stages, the evolution of crack morphologies was investigated using in-situ tensile test based on SEM (Fig. S2). It is found that at a low macro strain of 3.0%, local necking of hard Ti layer in LMC occurred, and dispersive micro-cracks appeared within the interface due to early incompatible deformation. [It](javascript:void(0);) [is](javascript:void(0);) [interesting](javascript:void(0);) [to](javascript:void(0);) [note](javascript:void(0);) that these local micro-cracks were steadily restricted in the interface and hardly propagated below a macro strain of 10.0%. As macro strain exceeded 10.0%, the local necking of hard Ti layer was hardly delayed by Al layer. As a result, these micro-cracks expanded and propagated along the interface. Once these interfacial cracks propagated to form the delamination, constraint effect disappeared locally. Then local large transverse cracks are easy to occur, resulting in the damage of LMC.


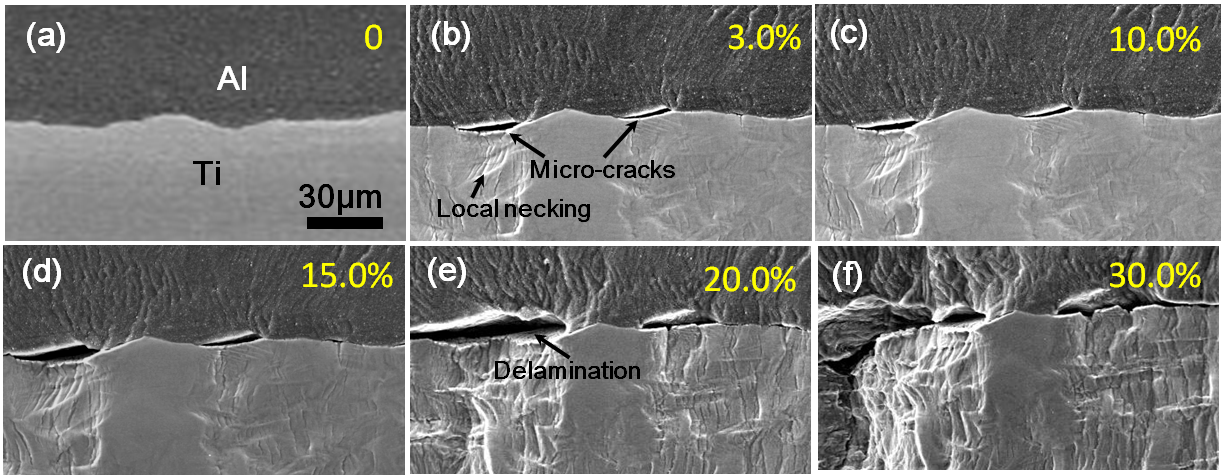


Fig. S2 The evolution of crack morphologies by in-situ tensile test based on SEM at different macro strains: (a) 0; (b) 3.0%; (c) 10.0%; (d) 15%; (e) 20%; (f) 30%.

***Part D Schematic of the hot rolling process of LMC***

The schematics of the fabrication processing of LMC were shown in Fig. S3. Firstly, Ti sheets and Al sheets were alternately placed and processed in vacuum at 500 ^o^C for 1 h with a pressure of 40 MPa to obtain well interface bonding. Then, LMC was rolled to a total thickness reduction of 50% after 6 rolling passes and annealed at 500 ^o^C for 10 minutes after each rolling pass.


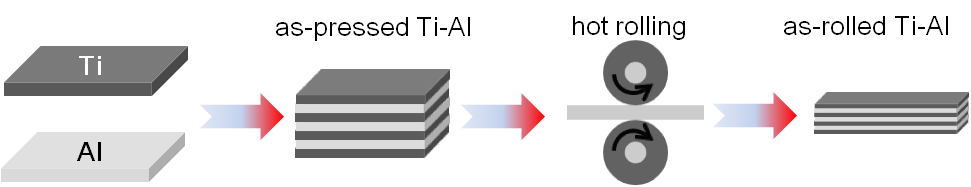


Fig. S3 Schematic of the hot rolling process of layered Ti-Al metal composite.

***Part E The experimental set-up and size of specimen for in-situ tensile test***

An interrupted tensile experiment was carried out using a Kammrath-Weiss micro-tensile stage, placed in the optical microscope (OM) as shown in Fig. S4a. For tensile testing half dog-bone shaped specimens were prepared to match with the dimension of the micro-tensile stage, following the geometry dimension (Fig. S4b).


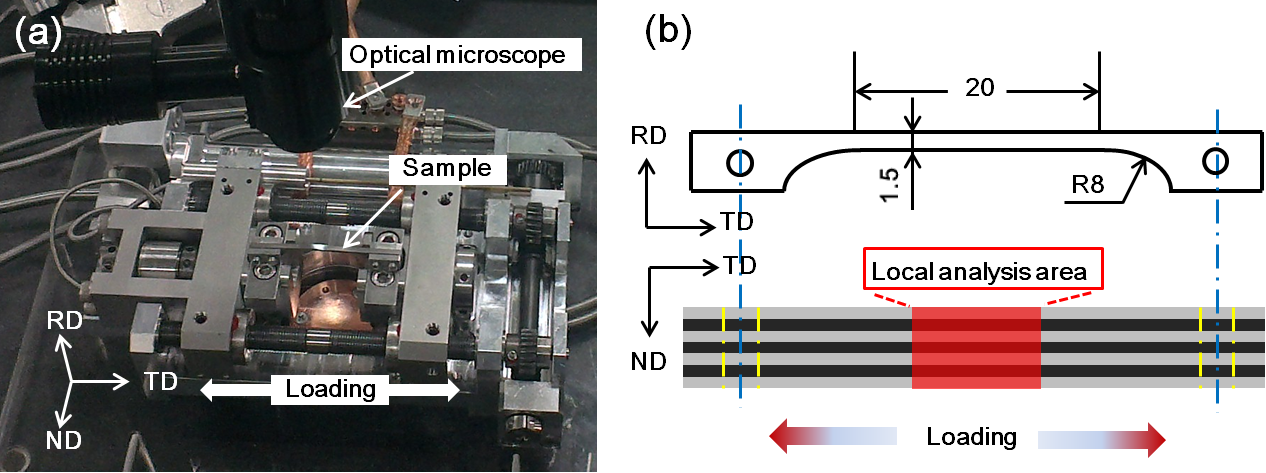


Fig. S4 (a) The experimental set-up; (b) size of specimen for in-situ tensile test.

***Part F Detail of X-ray tomography test***

The half of fractured tensile specimen was cut into cylindrical specimen with a diameter of 1 mm from middle area by electrical discharge machining, as shown in Fig. S5. Then the region closed to fracture within distance of 1 mm was selected as a testing region.


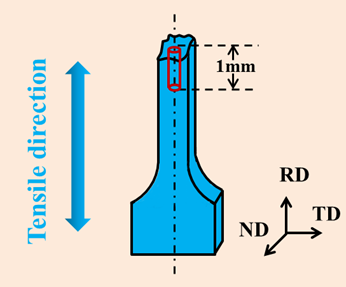


Fig. S5 Schematic drawing of specimen in X-ray tomography test.

The transmission beam of high energy X-ray irradiated the sample along the z axis direction from 0 to 180 °, and the original photos containing the information of 3D space were captured at each angle of rotation. Detailed test parameters are: X-ray energy (30 kev), the distance between samples and CCD (0.1 m), frequency of image acquisition (4s per sheet), resolution (0.65 μm per pixel), the size of collecting image (2048×2048 pixel^2^).

These original photos data were processed using the 3D analysis software (Avizo 9.0.1) and open source software (Image J), and then the 3D cracks distribution would be presented by the reconstruction of 2D slices (Fig. S6a and b). Based on the slice with different gray values (Fig. S6c), the crack area (*A*) of each slice is determined by corresponding gray value. The crack volume can be measured by the formula: *V_crack_ = Σ* (*A_i_* ×*Δd*) (where *A_i_* is the crack area in the number *i* of slice and *Δd* is the distance of adjacent slices with a constant value of 0.65 μm). The whole calculation process can be achieved by 3D analysis software (Avizo 9.0.1).

**
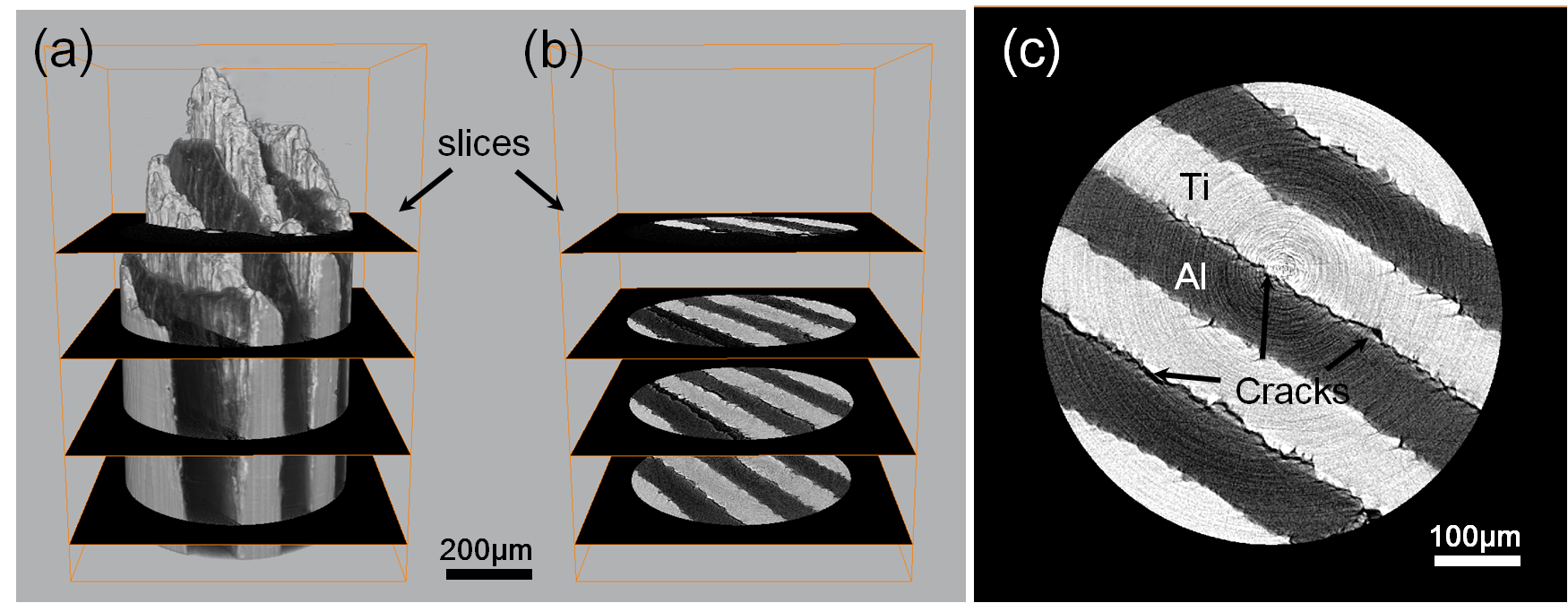
**

Fig. S6 (a) and (b) Schematic drawing of the reconstruction of 2D slices; (c) the crack area determined by different gray values in each slice. In (a) and (b), only four slices were schematically shown.
